# Axillary radiotherapy: an alternative treatment option for adjuvant axillary management of breast cancer

**DOI:** 10.1038/srep26304

**Published:** 2016-05-23

**Authors:** Jie Zhang, Chuan Wang

**Affiliations:** 1 Department of Breast Surgery, Affiliated Union Hospital of Fujian Medical University, Fuzhou, China

## Abstract

Axillary lymph node dissection is standard management of axilla in invasive breast cancer. Radiotherapy also is important in local treatment. It is controversial as to whether axillary radiotherapy can displace axillary lymph node dissection. We performed a meta-analysis comparing axillary radiotherapy with axillary dissection. No significant difference was observed for disease free survival and overall survival between the radiation group and the dissection group. There was also no significant difference in either the axillary recurrence or the local recurrence between the two groups. But the axillary relapse rate in the radiation group was higher than in the surgery group at five-year follow-up while the local recurrence rate in the surgery group was higher than in the radiation group. A subgroup analysis showed that the difference in the axillary recurrence rate (RR = 0.20, P = 0.01) and local recurrence rate (RR = 4.7, P = 0.01) mainly appeared in the clinical node-positive subgroup. The edema rate in the surgery group was higher than in the radiation group (RR = 2.08, 95%: 1.71–2.54, P < 0.0001). We concluded that radiotherapy may be an alternative treatment option for adjuvant management of the axilla in selected sub-groups of patients.

Axillary lymph node dissection has been considered the standard management of axilla in invasive breast cancer. It provides good control of local recurrence and provides information on axillary lymph node status which is a key factor of prognosis and guides further treatments. Nonetheless axillary lymph node dissection always induces functional sequelae, particularly lymphedema and restriction of shoulder mobility^1^,^2^. Sentinel node biopsy is regarded as standard surgical axillary management in clinical node-negative (cN0) breast cancer^3^. Many studies have proven the accuracy of a sentinel node biopsy for the assessment of lymph node status^4^,^5^. Those patients with pathologically negative sentinel nodes do not need to receive axillary lymph node dissection so that they will not suffer from excessive relative complications^6^,^7^. A sentinel node biopsy can provide enough predictive information on axillary lymph node status but fewer complications, so it has replaced axillary lymph node dissection and is now the preferred axillary surgical method in cN0 breast cancer patients. But should axillary lymph node dissection be performed if the sentinel lymph node is positive ? Recently, the ACOSOG Z0011 trial suggested that the patients who received breast-conserving surgery, whole breast radiotherapy and adjuvant systemic treatment may avoid axillary lymph node clearance if only one or two sentinel lymph nodes were positive^8^,^9^. The risks of local recurrence and the benefits of survival seemed not to be changed without axillary lymph node clearance in the subgroup of patients defined in the Z0011 study. However this study has a number of limitations, including failure to recruit a pre-specified number of patients to demonstrate equivalence, missing pathology details and uncertainty regarding the type of radiotherapy received and so on. The American Society of Clinical Oncology (ASCO) changed their guidelines following the publication of the Z0011 study^10^. At the same time, IBCSG 23-01, a phase III randomized controlled trial which focused on the patients with one or more micro-metastasis in sentinel lymph nodes, drew the same conclusion as Z0011^11^. But for the patients with more than two positive sentinel nodes or the patients who cannot receive a sentinel node biopsy, axillary lymph node dissection, at present, remains the standard of care^10^. The question remains as to whether other oncological treatments can replace axillary surgery for node positive patients who do not fit the Z0011 criteria? We noticed that all of the patients in the Z0011 study and nearly ninety percent of the patients in the IBCSG 23-01 study received breast-conserving surgery and tangential field radiotherapy at the same time^9^,^11^. Radiation may complement inadequate surgery and reduces the risk of loco-regional recurrence and it increases breast cancer specific overall survival^12^,^13^. Current breast cancer management guidelines offer axillary radiotherapy (ART) as an alternative to axillary lymph node dissection (ALND) in those not suitable for ALND, but there are only a few studies comparing ALND to ART prior to the AMAROS study which was a randomized, multicenter, open-label, non-inferiority phase III trial^14^,^15^. The AMAROS showed that, compared with ALND, ART achieved good axillary control but less lymphadenopathy morbidity^16^. This encouraging result must be interrupted with a caution given that there was a short follow up time and small number of recurrence events. In order to explore the outcomes of AMAROS and the value of ART as an alternative to ALND, we have undertaken a meta-analysis of the available literature.

## Material and Methods

### Identification of studies

The electronic databases PubMed, Embase, Cochrane and online abstracts from the proceeding Annual Meetings of the American Society of Clinical Oncology and San Antonio Breast Cancer Symposium (SABCS) were searched comprehensively and systematically. The following search strategy was used: (axillary OR axilla) AND (dissection OR clearance OR surgery) AND (radiotherapy OR irradiation OR radiation) AND (breast OR mammary) AND (cancer OR carcinom* OR neoplasm). Furthermore, references of selected studies were manually searched. If more than one study was based on the same research topic or reported the same data, only the study with the highest quality was selected. The final search was updated on September 2015.

### Selection Criteria

The titles and abstracts of selected studies were independently reviewed by two reviewers. Disagreements on study inclusion or exclusion were resolved by consensus. The inclusion criteria was: (1) randomization to either axillary radiotherapy or axillary lymph node dissection directly; (2) prospective randomized trials; (3) provided sufficient data to calculate relative risk (RR) or hazard ratio (HR) with 95% confidence interval (95% CI) of DFS, OS and local recurrence (LR). Reviews, case reports, systematic reviews, non-prospective clinical studies and multiple publications derived from the same clinical study were excluded. The studies whose data was too limited were also excluded.

### Data Extraction

Two investigators extracted data from the included studies independently. First, we checked the titles or abstracts. Full-text articles of potentially eligible studies were retrieved for further assessment. We extracted the following data: title, first author name, journal name, year of acceptance for publication, number of patients randomly assigned and analyzed, patient characteristics (age, menstrual status, tumor staging, molecular subtype (if available), treatment regimens (including surgical procedures, radiation dose and systemic treatment regiments). The primary outcomes were DFS and OS, the secondary outcomes were LR, distant recurrence and edema rate.

### Statistical analysis

Hazard ratio (HR) is the most appropriate statistics for meta-analyses for the time-to-event data such as OS and DFS. But the HR with 95% CI for DFS and OS was only provided directly in one study^16^. Others were extracted by means of Engauge Digitizers 4.1 from the survival curves and calculated via the method described by Tierney. J. *et al*.^17^. HR and 95% CIs were pooled according to the inverse of variance method. HR < 1 means that axillary dissection is favorable. As a dichotomous variable, relative risks (RRs) of event number of axillary recurrence, LR, metastasis and edema were calculated both in the axillary radiation group (ART) and axillary dissection group (ALND). A heterogeneity test and a random-effect (DerSimomian and Laird method) model were used in case of heterogeneity between the included studies, whereas a fixed-effect model (Mantel-Haenszel’s method) was used in the absence of any heterogeneity. A heterogeneity assumption was assessed via χ^2^ and I^2^. P < 0.10 in χ^2^ test and I^2^ values > 50% were considered to be significant heterogeneity. Publication bias was assessed by funnel plots. All analyses were performed using Review Manager software (version 5.3).

## Results

### Characteristics of selected studies

**Figure 1:**
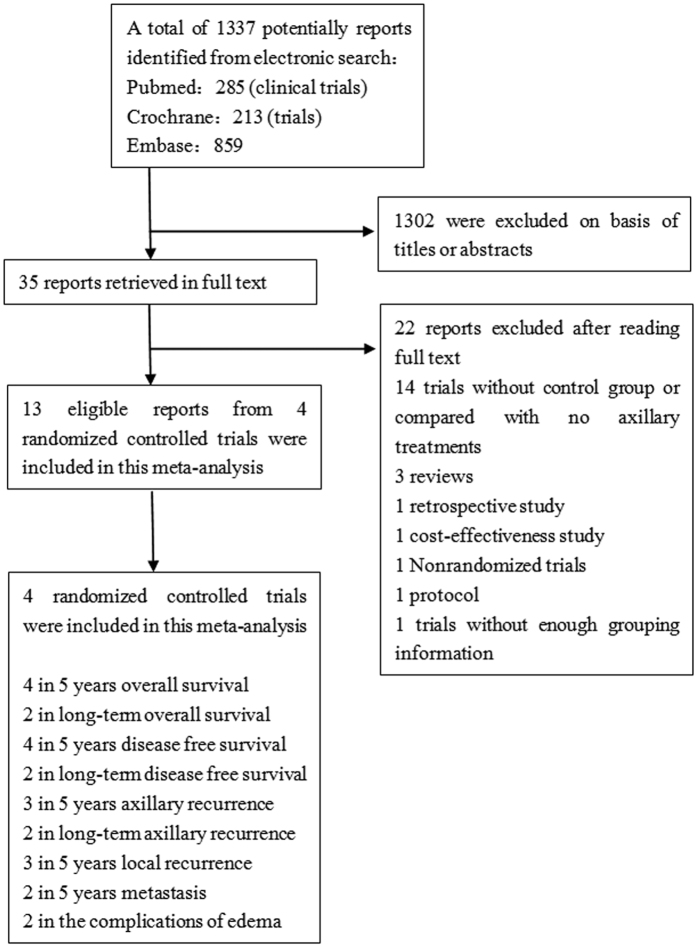
Flowchart of the study selection strategy.

The initial search identified 1,337 potentially relevant studies. After reviewing the titles and abstracts, 1,302 studies were excluded from the meta-analysis. The remaining 35 studies were considered eligible, and their full-text articles were reviewed. Of these, 13 reports from four randomized controlled trials that met the inclusion criteria were included in this meta-analysis. Finally, four randomized controlled trials were included in this meta-analysis. The process of study selection is summarized in Fig. 1. All of these four trials directly compared axillary dissection with axillary radiotherapy and enrolled 3,857 breast cancer patients. These patients were divided into two groups, 1,968 in the ALND group and 1,889 in the ART group. Nearly eighty-five percent of all patients recruited were clinical node-negative patients (1,676 in the ALND group and 1,595 in the ART group). All of the patients with clinically positive axillary lymph nodes came from the NSABP B-04 study (292 in the ALND group and 294 in the ART group)^14^,^18^,^19^. Sentinel lymph node biopsy (SLNB) was performed in two trials in which only the patients with pathological positive lymph node were included. The cN0 patients who were diagnosed as positive axillary lymph nodes by means of SLNB were assigned into a new subgroup (subgroup B). Among these trials, surgical procedures were breast conserving surgery (BCS) or mastectomy except for the NSABP B-04 study that performed radical mastectomy which is seldom used nowadays. Systemic treatments including endocrine therapy after local treatments were performed in all studies except the NSABP B-04 study. The primary purpose of NSABP B-04 was to compare “radical mastectomy” with “mastectomy” and with “mastectomy and radiotherapy”. It also compared ALND (Radical Mastectomy group) with ART (Mastectomy and radiotherapy group) at the same time. The patients‘ characteristics are well balanced between the two groups. Although NSABP B-04 which started patient entry in 1971 was considered an older study, and its treatments seem inappropriate today, it provides long-term results after 25 years of follow-up, while the follow-up time in the AMAROS and OTASOR studies as currently reported are no more than 5 years^16^,^20^. Characteristics of the included studies are listed in Table 1. None of the included trials provide information on the breast cancer subtypes.

### Primary outcome: OS and DFS

**Figure 2:**
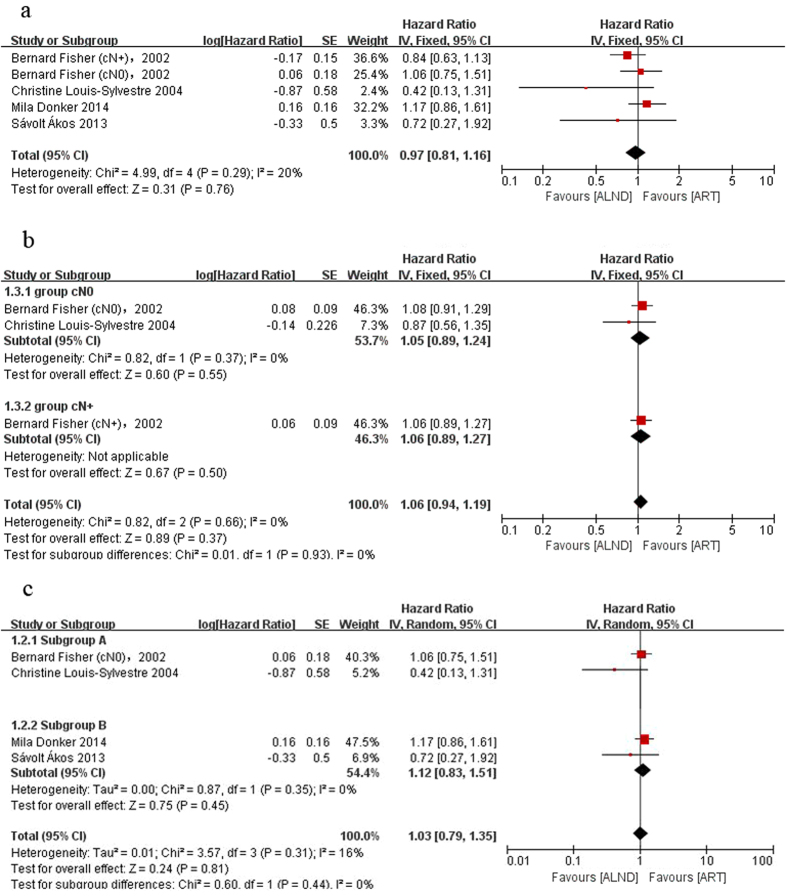
Forest plot of meta-analysis on overall survival (OS) of the axillary dissection group and axillary radiotherapy group. (**a**) Short-term outcome, (**b**) long-term outcome, (**c**) short-term outcome of the cN0 group.

**Figure 3:**
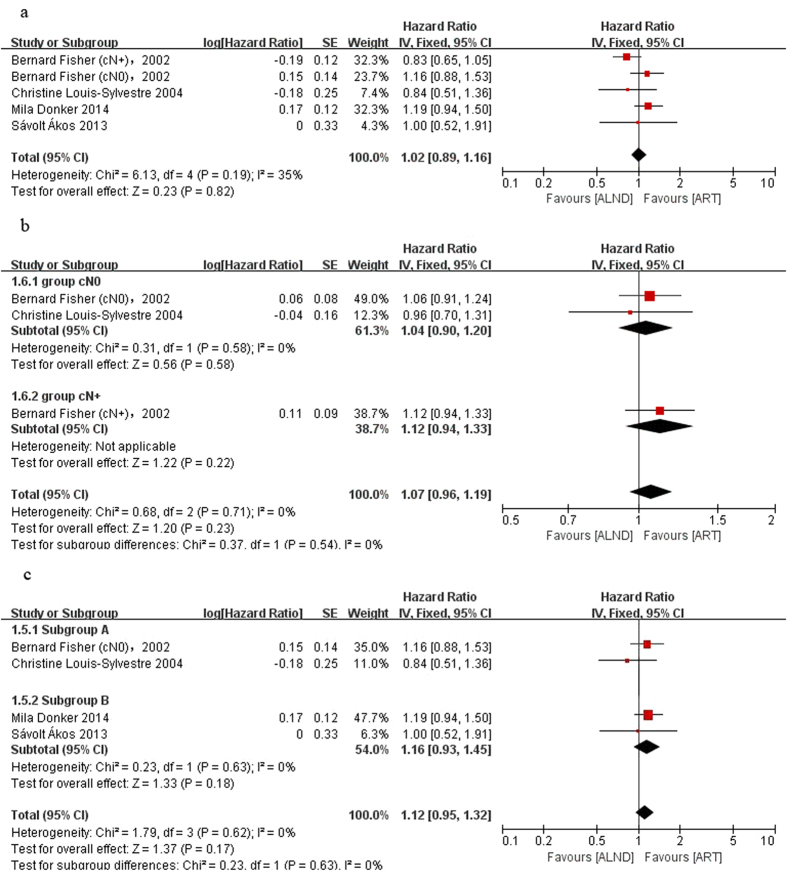
Forest plot of meta-analysis on disease free survival (DFS) of the axillary dissection group and axillary radiotherapy group. (**a**) Short-term outcome, (**b**) long-term outcome, (**c**) short-term outcome of the cN0 group.

We analyzed the primary data in seven reports from four trials. Since the follow-up time differed greatly, we analyzed the short term effect and long term effect separately. Five-year outcomes identified as short term survival data came from all the included trials and more than 15 years outcomes identified long term survival data that came from NSABP B-04 and Paris *et al*.^14^,^15^. The results showed that there was no significant difference between the two groups in either short term OS (HR = 0.97, 95% CI: 0.81–1.16, P = 0.76; Fig. 2a) and long term OS (HR = 1.06, 95% CI: 0.94–1.19, P = 0.37; Fig. 2b). The same results were found in both arms for disease free survival (short-term survival: HR = 1.02, 95% CI: 0.89–1.16, P = 0.82, Fig. 3a; long-term survival: HR = 1.07, 95% CI: 0.96–1.19, P = 0.23, Fig. 3b). Since the axillary lymph nodes of many patients in this analysis were clinically negative, we established an independent group which included these patients and assigned the patients who were diagnosed as positive axillary lymph nodes by means of SNB into a new subgroup (subgroup B, just cN0 pN+). There was still no significant difference between the ALND and ART groups in the subgroups. The HR for short-term OS is 1.03 (95% CI: 0.79–1.35; P = 0.81) in the cN0 group and 1.12 (95% CI: 0.83–1.51; P = 0.45) in the subgroup B (Fig. 2c). The HR for long-term OS is 1.05 (95% CI: 0.89–1.24; P = 0.55, Fig. 2b) in the cN0 group. The HR for short-term DFS is 1.12 (95% CI: 0.95–1.32; P = 0.17) in the cN0 group and 1.16 (95% CI: 0.93–1.45; P = 0.18) in the subgroup B (Fig. 3c). The HR for long-term DFS in cN0 patients is 1.04 (95% CI: 0.9–1.21; P = 0.58, Fig. 3b). No significant heterogeneity was observed. These findings suggest that ART may achieve the same survival outcomes compared with ALND.

### Secondary outcome: axillary recurrence, local recurrence, distant recurrence and complications

**Figure 4:**
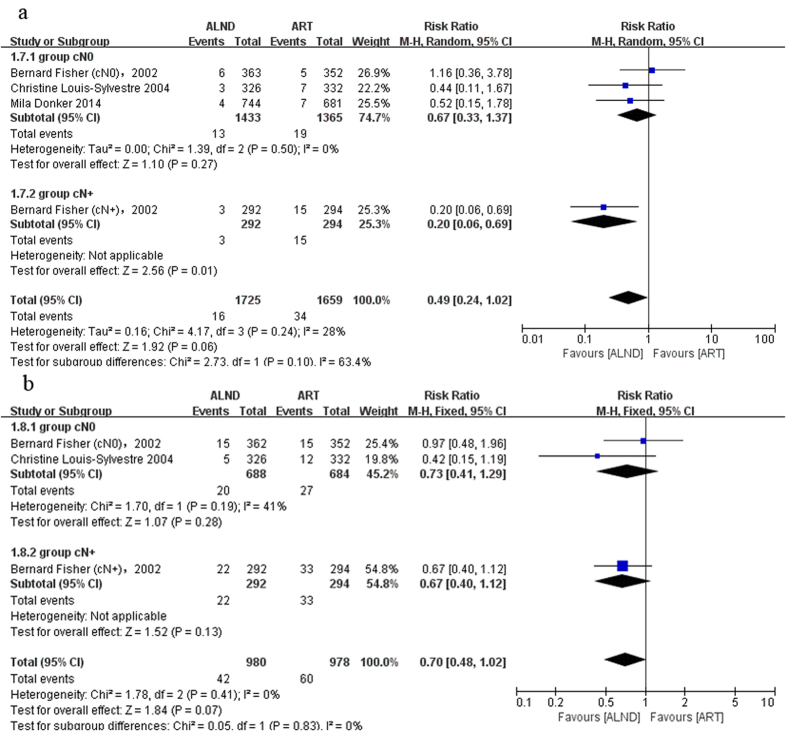
Forest plot of meta-analysis on axillary recurrence (AR) of the axillary dissection group and axillary radiotherapy group. (**a**) Short-term outcome, (**b**) long-term outcome.

**Figure 5:**
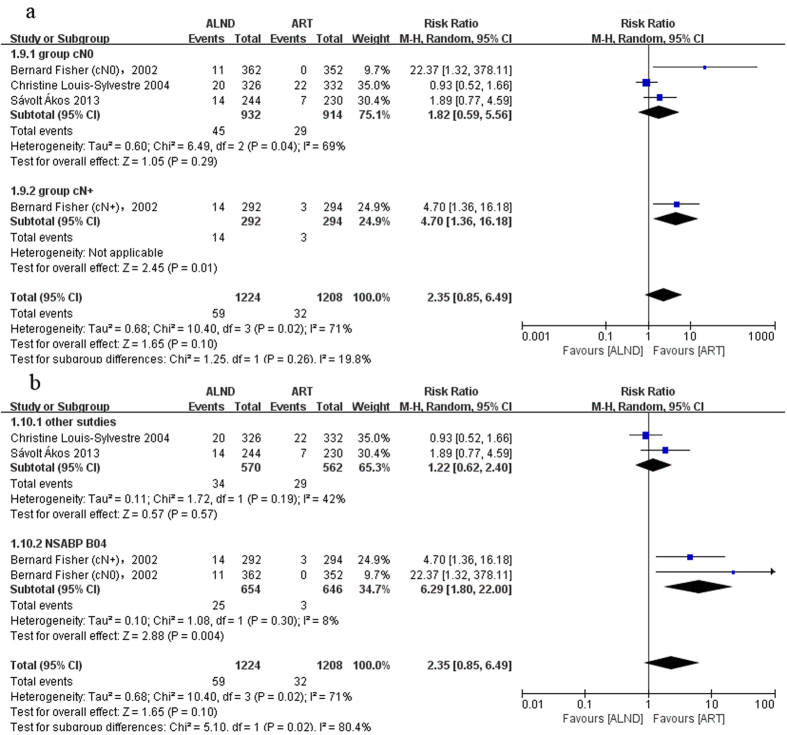
Forest plot of meta-analysis on local recurrence (LR) of the axillary dissection group and axillary radiotherapy group. (**a**) Short-term outcome including the cN0 group and the cN+ group, (**b**) Short-term outcome including subgroup analysis for exploring the source of heterogeneity.

The axillary recurrence was defined as axillary lymph node recurrence, but there were no specific events of axillary lymph node recurrence in the NSABP B-04. Thus we used the events of regional node recurrence to replace it. Local recurrence meant relapse occurred in the place limited to the breast or chest wall. Short-term recurrence rate was reported in three trials. But only one trial provided long-term local recurrence results. Because of the limited data, it was not possible to undertake the analysis of long-term outcomes. The results showed that the axillary relapse rate in the radiation group was higher without statistical significance in short term follow-up (RR = 0.49, 95%: 0.24–1.02, P = 0.06) (Fig. 4a). In a longer follow-up period (more than 15 years), the tendency did not change (RR = 0.70, 95%: 0.48–1.02, P = 0.07) (Fig. 4b). A subgroup analysis showed that the difference was mainly attributed to the cN+ subgroup especially in the short-term follow-up (short-term follow-up: RR = 0.20, 95%: 0.06–0.69, P = 0.01, long-term follow-up: RR = 0.67, 95%: 0.40–1.12, P = 0.13) (Fig. 4). The axillary relapse rate did not differ significantly in both groups in the cN0 subgroup (short-term follow-up: RR = 0.67, 95%: 0.33–1.37, P = 0.27; long-term follow-up: RR = 0.73, 95%: 0.41–1.29, P = 0.28) (Fig. 4). The heterogeneity was significant between the cN0 and cN+ subgroups (P = 0.10, I^2^ = 63.4) (Fig. 4a), which confirmed the above speculation. On the contrary, the local recurrence rate was higher in the surgery group (Fig. 5). Since significant heterogeneity was found in the cN0 subgroup, a random-effects model was used. The difference in the number of local recurrences between the ART and ALND groups was not statistically significant (overall: RR = 2.35, 95%: 0.85–6.49, P = 0.10; cN0 subgroup: RR = 1.82, 95%: 0.59–5.56, P = 0.29; Fig. 5a), except for the cN+ subgroup which was reported by only one clinical trial (RR = 4.70, 95%: 1.36–16.18, P = 0.01; Fig. 5a). Subgroup analysis revealed that the heterogeneity mainly came from the NSABP B-04 trial. No difference was found after excluding this trial (P = 0.57; Fig. 5b).

**Figure 6:**
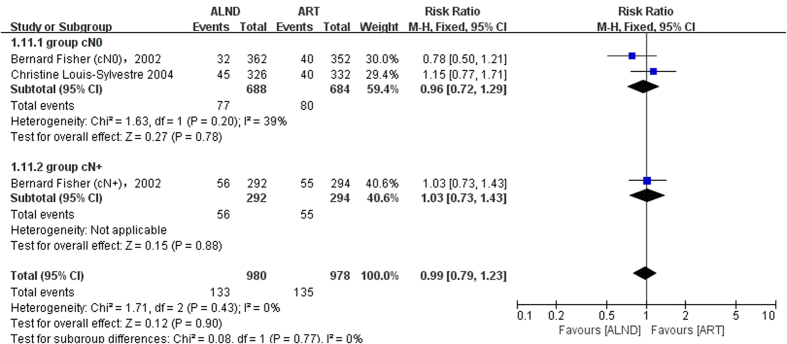
Forest plot of meta-analysis on short outcome of distant recurrence (DR) of the axillary dissection group and axillary radiotherapy group.

Only two trials reported short-term outcomes about distant metastases. The results showed that there was no difference between the radiation and surgery groups (overall: P = 0.90, cN0 subgroup: P = 0.78; Fig. 6).

**Figure 7:**
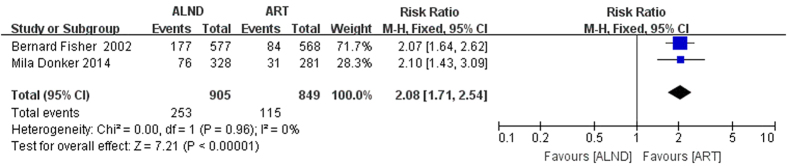
Forest plot of meta-analysis of lymphoedema risk of the axillary dissection group and axillary radiotherapy group.

Lymphoedema in the ipsilateral arm is the most common sequelae after axillary treatment and often causes severe distending pain and dysfunction. Data about lymphoedema was collected from two trials: the AMAROS trial which was followed up for five years and the NSABP B-04 trial which was followed up for twenty years^2^,^16^. For the NSABP B-04, the incidence of edema was extracted from the final submitted arm measurement form. The patients who had arm edema on at least one measurement during the follow-up period but finally recovered were excluded from analysis, 1754 patients were included in this assessment. There were 253 out of 905 (28.0%) patients in the ALND group and 115 out of 849 (13.5%) patients in ART group that suffered from arm lymphedema. The difference was statistically significant (RR = 2.08, 95% CI: 1.71–2.54, P < 0.00001; Fig. 7).

## Discussion

Our meta-analysis was based upon four trials. The OS and DFS were not significantly different between axillary dissection and axillary radiation in short or long term outcomes. Therefore we suggest that axillary radiation may be another choice of axillary treatment in terms of survival. This result was consistent with each trial included in our study^14^,^15^,^16^,^20^. But only one different conclusion was presented in the 5-years outcomes of a randomized study by Paris *et al*.^21^. An improved survival rate in the axillary dissection group was observed compared with axillary radiation. They considered the reason for the difference was that more patients in the ALND group were treated with adjuvant chemotherapy. There are nine patients in the ART group who received systemic treatments, whereas there are nineteen patients in the ALND group. But this influence did not last until longer follow up. The other trial that presented long term outcomes is NSABP B-04. It is the earliest randomized clinical trial that compared axillary lymph node dissection with axillary radiation directly^18^. After 25 years follow up, there was no significant difference in the DFS and OS in the two groups^14^. We should consider that the survival benefited mainly from local treatment because no systemic treatment was performed in this trial. So the NSABP B-04 provided stronger evidence that axillary radiation would not lead to different survival compared with axillary dissection without the influence of systemic therapy. The Early Breast Cancer Trialists’ Collaborative Group (EBCTCG) reported a meta-analysis which also showed that early breast cancer patients who received postoperative radiotherapy could gain 1.2% more in their overall survival rate^22^.

On the other hand, we must also consider that effective systemic therapy might reduce breast cancer mortality by around one-third^23^. Effective systemic therapies including chemotherapy, endocrine therapy, targeted therapy which was carried out in the clinical trial OTOASOR and AMAROS may bring about a better survival rate and weaken the effect of local treatment on long-term survival. Therefore different local treatment mainly affects the local-regional recurrence rate. In our study, we found there were no statistical differences between the ALND group and the ART group whether in axillary recurrence or in local recurrence. But the ALND group had a higher local recurrence rate than the ART group. At the same time a significant heterogeneity was found in analysis of local recurrence. And the heterogeneity mainly came from the NSABP B-04 which presented a significant difference of local recurrence between the two groups, otherwise no difference was found after making a conjoint analysis on the other two trials. We considered that systemic therapy may be a key factor which covered up the effect caused by local treatment. Since the contribution of axillary radiation on reducing the local recurrence rate was easily observed in the NSABP B-04 in which there was no systemic therapy effect. The axillary radiation which covered the chest wall may contribute to the local control. This supposition was supported by the MA.20 clinical trial which found that adding regional nodal radiation to whole-breast radiation may reduce local recurrence rates^24^. On the contrary the axillary recurrence rate of the radiation group was obviously higher than the surgery group both in short-term outcomes and long-term outcomes, although no statistically significant difference was found. A retrospective study confirmed this result. They found 10-year cumulative axillary recurrence rates were 1.3% and 4.6% for the ALND group and the ART group respectively without a statistically significant difference^25^. This reaffirms that axillary radiation does not bring about complete axillary control for all patients. In our study, the further subgroup analysis showed that the difference of axillary recurrence was mainly attributed to the cN+ subgroup not the cN0 subgroup. This result was in accordance with some other trials. Veronesi *et al*. found that axillary recurrence rate was low (1 in 221) in the patients who did not have palpable axillary lymph nodes and treated with breast conservation plus axillary radiation after 63 months follow-up^26^. Another result reported by Frank J. *et al*. also showed a low axillary recurrence rate (about 2%) in the cN0 patients who received wide local excision and radiotherapy after 44 months follow-up^27^. These studies suggest that patient characteristics are also important in determining axillary outcome. Axillary radiation might be a safety choice among the patients with clinical-negative axillary lymph nodes. Effective local treatments lead to a reduction in distant recurrence^12^. No difference in the rate of distant metastases was found in the ALND group and the ART group in our study both in the cN0 patients and the cN+ patients.

In current practice, overall survival of breast cancer patients has been improved because of progress in diagnostic techniques and systemic treatments^23^,^28^. Since the prognosis of breast cancer patients is relatively better, a better life quality was accordingly demanded. But axillary lymph node dissection is always associated with harmful and persistent complications, such as lymphedema. And patients would gain much better life quality without axillary dissection^7^,^29^. Some studies showed that the rate of complications seemed to be the same for the two kinds of axillary treatments. They reported that the arm edema rate was 10% after axillary surgery and approximately the same percentage after axillary radiation^27^,^30^,^31^. In our study, we found that the edema rate caused by axillary dissection was about 30% which is much higher than the result published before. We consider that the operation method and follow-up time might be the main reason. The mean number of lymph nodes removed was few (only four) and the follow-up time was too short (38.5 months) in the previous studies^31^. But the edema rate after axillary radiation in our study was about 13%, which is similar to the previous studies^27^. Radiation reduced the edema rate by 50% compared with axillary surgery. So we concluded that axillary radiation induces fewer side-effects than axillary dissection, which can greatly improve life quality.

Our meta-analysis of the current available studies demonstrates that axillary radiation may have the same “curative” effect but induces fewer complications compared with axillary surgery. And who may benefit from it? The subgroup analysis showed that there was no difference between radiation and dissection on survival and recurrence rates in the cN0 patients. But the very important information of axillary staging would be missed if axillary dissection is avoided. Nowadays a sentinel node biopsy becomes the standard treatment for the cN0 breast cancer patients. It may give us the same information as axillary dissection. Then, avoiding axillary surgery in clinical node-negative but pathological node-positive patients is now feasible and worthwhile. Our meta-analysis included two trials which enrolled cN0 pN+ patients. The subgroup analysis showed that there was no difference of overall survival, disease free survival and regional-local recurrence in the cN0 pN+ patients. Radiation may also be a good choice for those patients other than surgery. But for the patients with high recurrence and metastasis risks, such as the cN+ patients whose lymph nodes are palpable, only one method of local treatment seemed to be not enough. As the EBCTCG shows, surgery and local radiation must be combined to reduce local recurrence and extend survival^13^,^22^.

We must report there are some limitations in our meta-analysis. 1) Because only four trials focused on this topic for some conclusions there are too few events based on limited data that limit the strength of our conclusions. 2) None of these articles provides data about different subtypes of breast cancer. We are not completely confident about whether axillary radiation is sufficient or not when we consider treating some patients with a poor prognosis subtype, such as triple negative subtype. So, whether axillary radiation can become an effective management of the axilla or not is still uncertain. We hope our article can serve as a modest spur to encourage more in-depth studies to resolve this area.

## Additional Information

**How to cite this article**: Zhang, J. and Wang, C. Axillary radiotherapy: an alternative treatment option for adjuvant axillary management of breast cancer. *Sci. Rep.* **6**, 26304; doi: 10.1038/srep26304 (2016).

## Figures and Tables

**Table 1:** Main characteristics of the included studies.

**Study**	**Years**	**Intervention**	**Lymph node status**	**Follow-up time**	**No. of patients**
NSABP B-04^14^,^18^,^19^ (Bernard Fisher)	2002	Radical mastectomy	cN + pN(NA)	25 years	292
		Total mastectomy + radiotherapy	cN + pN(NA)		294
	2002	Radical mastectomy	cN0 pN(NA)		362
		Total mastectomy + radiotherapy	cN0 pN(NA)		352
Paris^15^,^21^ (Louis-Sylvestre)	2004	Lumpectomy + axillary dissection	cN0 pN(NA)	15 years	326
		Lumpectomy + axillary radiotherapy	cN0 pN(NA)		332
OTOASOR^20^ (Sávolt Ákos)	2013	Breast surgery + axillary dissection	cN0 pN+	40 months	244
		Breast surgery + axillary radiotherapy	cN0 pN+		230
AMAROS^16^,^32^ (Mila Donker)	2014	Breast surgery + axillary dissection	cN0 pN+	6.1 years	744
		Breast surgery + axillary radiotherapy	cN0 pN+		681

NA: not applicable, cN0: clinical node-negative, cN+: clinical node-positive, pN: pathological axillary lymph node status.
